# Sustainable energy storage: *Mangifera indica* leaf waste-derived activated carbon for long-life, high-performance supercapacitors†

**DOI:** 10.1039/d3ra08910j

**Published:** 2024-03-07

**Authors:** Shreeganesh Subraya Hegde, Badekai Ramachandra Bhat

**Affiliations:** a Catalysis and Materials Chemistry Laboratory, Department of Chemistry, National Institute of Technology Karnataka Surathkal Mangalore 575025 Karnataka India hegdeshreeganesh@gmail.com ram@nitk.edu.in

## Abstract

Biomass waste-derived activated carbon has a wide range of applications, including air and water purification, gas separation, energy storage, and catalysis. This material has become increasingly popular in recent years as a result of the growing demand for sustainable and eco-friendly materials. In this study, *Mangifera indica* leaf waste-derived activated carbon has been investigated as an electrode material for high-performance supercapacitors. The dried *Mangifera indica* leaves were first carbonized using FeCl_3_ and then activated using KOH to increase their surface area and pore structure at different temperatures. The activated carbon prepared at 725 °C has shown a high specific capacitance of 521.65 F g^−1^ at a current density of 0.5 A g^−1^ and also achieved an energy density of 17.04 W h kg^−1^ at a power density of 242.50 W kg^−1^ in the 6 M KOH electrolyte. Significantly, it has demonstrated remarkable electrochemical cycling stability, retaining 96.60% of its initial capacity even after undergoing 10 001 cycles at a scan rate of 500 mV s^−1^. The superior electrochemical performance of the activated carbon can be attributed to its high surface area of 1232.63 m^2^ g^−1^, well-distributed pore size, and excellent degree of graphitization, which all facilitate the rapid diffusion of ions and enhance the accessibility of the electrolyte to the electrode surface. Hence, this study provides a promising route for utilizing waste biomass as a low-cost, sustainable electrode material for energy storage devices.

## Abstract 2

This study provides a promising route for utilizing waste biomass as a low-cost, sustainable electrode material for energy storage devices.

## Graphical abstract

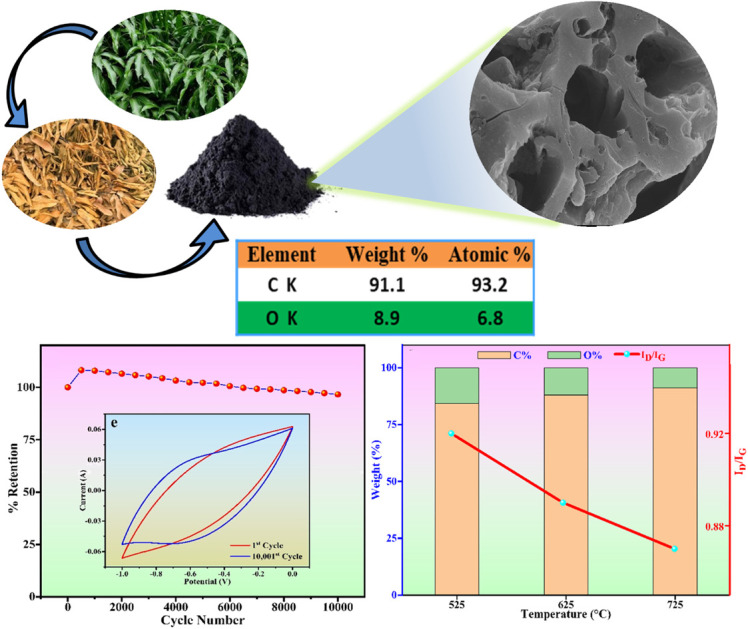

## 1. Introduction

With the overuse and exploitation of fossil fuels like coal and oil, contemporary world civilization has been confronted with a growing number of significant energy problems and environmental degradation.^1,2^ Hence, the majority of nations in the world have developed double-carbon policies that made great efforts to create and employ green, renewable resources in order to address the aforementioned issues with sustaining rapid economic development.^3^ Recent investigations on the production of activated porous carbon from environmental waste and its usage for various applications have drawn much scientific attention.^4^ At the same time, creating new carbon materials with large-scale applications must adhere to industrial requirements such as environmental sustainability, an inexpensive or simple production method, and the disclosure of enhanced or even novel desired features.^5,6^ In addition to their superior chemical and thermal stability, the high surface area, variable porosity, and pore sizes of these activated or porous carbons have particularly gained interest.^7^ These conditions are satisfied by porous activated carbons made from inexpensive environmental waste precursors, particularly biomass. Most significantly, biomass is ideally suited for the preparation of carbon electrode materials for energy storage devices like supercapacitors due to its extremely high percentage of carbon content and unique physiochemical properties.^8^

Carbon materials are electrically conductive, have a low electrical resistance, a large specific surface area, and can physically adsorb a lot of charges onto their surface. Because of this, supercapacitors made of carbon-based materials frequently have a good specific capacitance, an extended lifespan, and excellent performance stability.^9–12^ However, carbon nanotubes, graphene materials, and fullerene are the most appreciated carbon-based electrode materials but typically have complicated synthetic processes and expensive manufacturing costs, making them unsuitable for widespread commercial use.^13^ As a result of their simplicity in synthesis, low cost, flexible pore designs, and excellent chemical and thermal stability, porous activated carbon materials show tremendous potential in this respect.^7^ Food wastes, municipal solid wastes, agricultural and animal wastes, and other environmental wastes have been widely used in the creation of porous activated carbon through carbonization and activation processes. The characteristics of these porous activated carbons can be regulated by adjusting the activator/carbon precursor ratio, activation pyrolysis temperature, and activating agents like ZnCl_2_, FeCl_3_, H_3_PO_4_, K_2_CO_3_, KOH, *etc.*^14,15^

In the current study, we thoroughly investigated how activating temperature affected the porous texture, surface characteristics, and electrochemical performance of activated carbon. Here, dead *Mangifera indica* leaves waste was employed as the feedstock, and porous activated carbon was synthesized using a two-step process with changing temperatures. X-ray diffraction (XRD), Raman spectroscopy, FTIR, field-emission scanning electron microscopy (FESEM), EDX, TEM, XPS, and BET investigations have been used to study the different physicochemical aspects of the produced material. Finally, the electrochemical performance of synthesized activated carbon materials was assessed as electrodes for the supercapacitors.

## 2. Materials and methods

### 2.1. Materials

Potassium hydroxide pellets, ferric chloride, and hydrochloric acid were purchased from Loba Chemie. Absolute ethanol was purchased from Changshu Hongsheng Fine Chemicals, *N*-methyl-2-pyrrolidone (NMP), and poly(vinylidene fluoride) (PVDF) were purchased from Sigma-Aldrich, Grafoil sheets, TIMCAL C-NERGY SUPER C65 were purchased and were used as-received. Type 1 ultrapure water (Elga Veolia) was used throughout the experiments to prepare all the solutions. Dead *Mangifera indica* leaves (DML) was collected near Birangod, Honnavara, Uttarakannada, Karnataka and used as a precursor material to prepare *Mangifera indica* leaves-derived activated carbon (MLAC).

### 2.2. Synthesis of *Mangifera indica* leaves derived activated carbon (MLAC)

Dead *Mangifera indica* leaves (DML) were sun dried for a week and grounded to make powder. Then it was washed several times thoroughly with ultrapure water followed by ethanol, dried it in an oven and finally pulverized. Preparation of MLAC from DML was carried out in two-step process. Using FeCl_3_, the carbonization of DML was done in the first step, followed by chemical activation using KOH in the second step. In the first step, 1 : 1 ratio of DML was mixed with FeCl_3_ in minimum amount of ultrapure water and stirred using a magnetic bar at about 100 °C until it became a solid paste, and then it was placed in a hot air oven at 110 °C for about 24–48 h. Then FeCl_3_-treated DML was carbonized in an argon gas environment at 400 °C for 1.5 h. In the second step, the FeCl_3_-treated DML powder sample was mixed with 10 g of KOH in ultrapure water with continuous stirring on a hotplate to make it solid paste, and then it was dried in a hot air oven at about 110 °C for 24–48 h. The KOH-treated DML was pyrolyzed in a silica boat at various temperatures, including 525 °C, 625 °C, and 725 °C for 90 minutes in a continuous argon environment at rapid heating rate in a tube furnace to determine the optimal activation temperature. The carbonized samples were poured into a diluted HCl solution and then rinsed with deionized water until it reached neutral pH to effectively remove the residual FeCl_3_, KOH, and inherent ash minerals. Finally, these samples were treated with ethanol followed by centrifugation. Before determining their morphological and electrochemical properties, the resulting products (denoted as MLAC-500, MLAC-600, and MLAC-700 respectively) were dried at about 110 °C for 24–48 h. The yield of carbonized DML, MLAC-525, MLAC-625, and MLAC-725 was found to be 16 g, 2.1 g, 1.9 g and 1.6 g respectively.

### 2.3. Characterizations

X-ray diffraction (XRD) studies of the synthesized samples were carried out using monochromatic Cu Kα radiation of a wavelength of 0.154 nm on Malvern PANalytical (Empyrean 3rd Gen, Netherlands) instrument. Raman analysis was done using Compact Raman spectrometer (Renishaw, UK). FTIR spectrum was recorded by Spectrum Two FT-IR spectrometer (PerkinElmer, Singapore) in the spectral range of 500–4000 cm^−1^. The energy dispersive X-ray (EDX) analysis and the field emission scanning electron microscopy (FESEM) images were taken by Jeol (Japan) instrument. Transmission electron microscope (TEM) images were taken using the JEOL JEM-2100. X-Ray Photoelectron Spectroscopy (XPS) was used to confirm the elemental composition in the sample. The specific surface area, pore characteristics of the synthesized materials and N_2_ adsorption–desorption studies were carried out using an Autosorb (Anton Paar IQ-XR-XR, Austria) instrument. Before the BET measurement, samples were degassed at 300 °C in a vacuum for three hours.

### 2.4. Electrode preparation and electrochemical characterizations

The working electrodes were prepared by drop-casting the NMP solvent-based mixture of 80% MLAC, 10% C-NERGY SUPER C65 and 10% PVDF binders on a 1 × 1 cm^2^ Grafoil sheet. The mass loading of the active material on the electrode was 3.00 mg cm^−2^. These electrodes were dried in a vacuum oven at 60 °C for overnight. All the electrochemical measurements were carried out in two symmetric electrode system on an electrochemical workstation (Autolab) using a 6 M KOH electrolyte at room temperature. The cyclic voltammetry (CV) and galvanostatic charge–discharge (GCD) measurements were carried out in the potential range from −1 V to 0 V at various scan rates ranging from 10 mV s^−1^ to 200 mV s^−1^ and current densities ranging from 0.5 to 6 A g^−1^, respectively. The electrochemical impedance spectroscopy (EIS) measurements were carried out in the 10 mHz to 100 kHz range with an amplitude of 10 mV. The cyclic stability of the electrodes was studied using the CV technique at a scan rate of 500 mV s^−1^ for up to 10 001 cycles. From the charge–discharge measurements, the capacitance (*C*, F g^−1^) of the single electrode, energy density (*E*, W h kg^−1^) and the power density (*P*, W kg^−1^) were calculated respectively from following equations;^16^
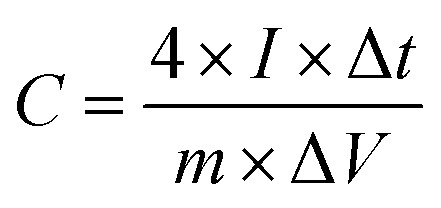

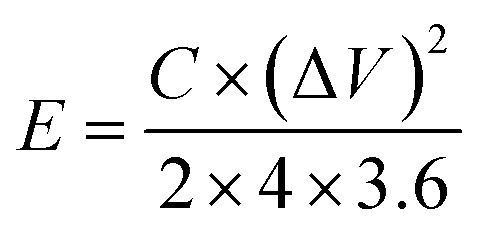

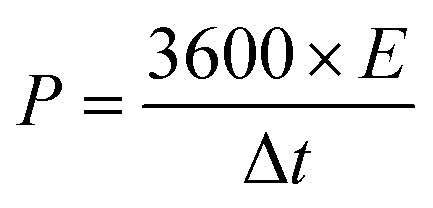
where *I* (A) is the constant discharge current, Δ*t* (s) is the discharge time, *m* (g) is the mass of active material on single electrode, and Δ*V* (V) is the potential change.

## 3. Results and discussions

### 3.1. Structural and morphological analysis

**Fig. 1:**
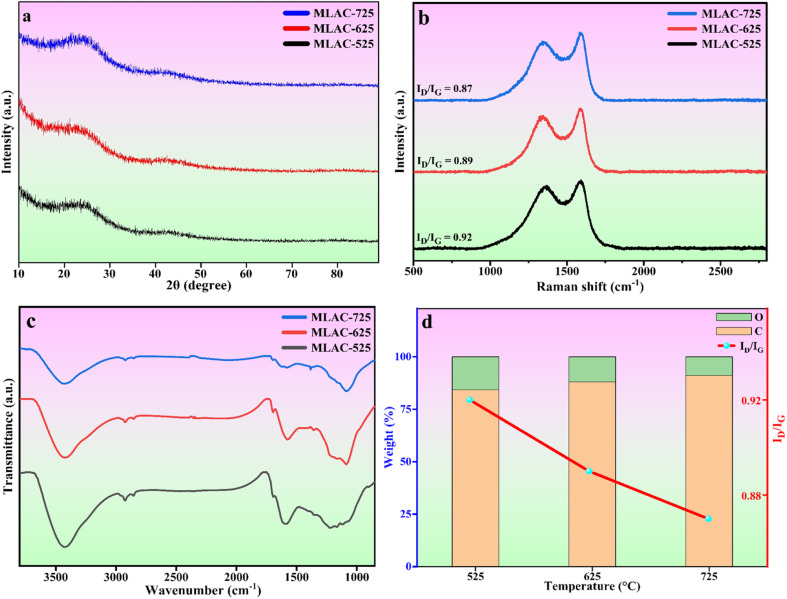
(a) XRD patterns, (b) Raman spectra, (c) FTIR spectra, (d) effect of temperature on elemental weight% and Raman intensity ratio.

The crystal structures of MLAC-525, MLAC-625, and MLAC-725 were investigated using the X-ray diffraction technique (XRD), and the relevant XRD patterns are very similar, as shown in Fig. 1a. The XRD spectrum of MLACs exhibits two typical prominent broad peaks, ∼26° and ∼43°, due to the presence of (002) and (100) crystal planes, respectively.^16^ The broadness of the peaks indicates that the activated carbon compounds have amorphous structures and low levels of graphitization. But as the pyrolysis temperature of MLAC raised from 525 °C to 725 °C, the intensity of the peaks increased and decrement in broadness, suggesting the improvement in graphitization. Further, this improvement in graphitization can be supported by Raman analysis.

Raman spectroscopy, a non-destructive characterization method, was used to examine the extent of changes in the order of the arrangement of carbon structures in MLAC-525, MLAC-625, and MLAC-725 during the graphitization phase and also to figure out the microstructural changes that occurred during the activation. The D-band, which originates from disordered carbon, can be seen in all Raman spectra (Fig. 1b) of materials between 1339 cm^−1^ and 1364 cm^−1^, while the characteristic G-bond, which comes from sp^2^-hybridized graphitic carbon, can be seen between 1581 cm^−1^ and 1591 cm^−1^. A commonly utilized method to assess the degree of disorder in carbon structures involves calculating the intensity ratio of two distinct bands, referred to as *I*_D_/*I*_G_. The peak intensity of XRD and the *I*_D_/*I*_G_ value in the Raman spectra are measures of graphitic order or crystallinity in the activated carbon and are inversely related. The increase in peak intensity in the XRD pattern is a result of an improvement in crystallinity or the development of graphitic orders, which leads to a decrease in the value of the *I*_D_/*I*_G_ ratio in the Raman spectra.^17^ The *I*_G_/*I*_D_ values of MLAC-525, MLAC-625, and MLAC-725 are 0.92, 0.89, and 0.87, respectively. Fig. 1d portrays the relation between the graphitization temperature and the *I*_D_/*I*_G_ values. When the graphitization temperature of MLAC is raised from 525 °C to 725 °C, the strength of the G band increases while the intensity of the D band decreases, suggesting a growth in the graphitic content with a decrease in defects.^18^ The degree of graphitization or defect density of activated carbon is crucial in enhancing its electrical characteristics when using it to make supercapacitors. The proper coexistence of amorphous and graphitic carbon could enhance the active area on the surface, conductivity, and wettability, all of which would be advantageous for improving capacitance.^19–22^

Fourier-transform infrared spectroscopy (FTIR) was used to examine the functional groups of MLAC, as shown in Fig. 1c. Based on the pertinent literature,^23–26^ the band at 3430 cm^−1^ is associated with the hydroxyl molecule (O–H). The presence of an aliphatic C–H framework, which includes CH_3_ and CH_2_ in the alkyl group, corresponds to the bands at 2922 cm^−1^. The functional groups C=O and C=C are attributed to the 1696 cm^−1^ and 1590 cm^−1^ bands, respectively. C–H bending vibration is responsible for the band at 1368 cm^−1^. The stretching vibration of the C–O can be used to explain the bands at 1094 cm^−1^. The FTIR spectra of the MLAC showed changes in transmittance as the pyrolysis temperature increased. With the rising preparation temperature of MLAC from 525 °C to 725 °C, the peak at around 3430 cm^−1^ showed a constant reduction in intensity, suggesting the loss of hydroxyl groups. The band at 2922 cm^−1^ had a similar tendency to that of the hydroxyl band, with its strength quickly declining with increasing temperature due to the virtually disappearing C–H bonds. The aliphatic bands show the progressive eradication of the aliphatic structures, which gradually decrease as the temperature increases. In light of this, it is anticipated that the resulting preparation temperature will cause the rearrangement of the carbon skeleton due to the decomposition and breaking down of the aliphatic framework during activation. In conclusion, the primary chemical modifications that occurred during pyrolysis included dehydration, formation and elimination of carbonyl group, breaking of aliphatic side chains, and creation of aromatic carbon groups.^26^

**Fig. 2:**
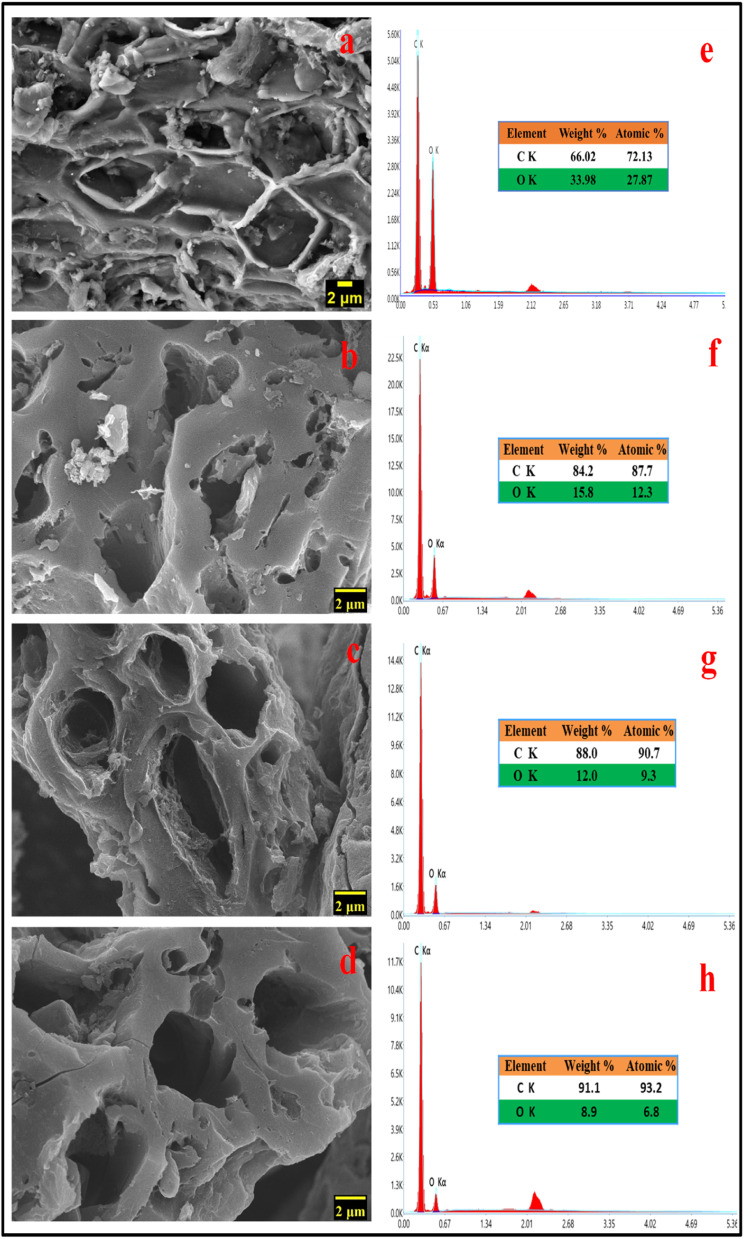
FESEM images of (a) carbonized DML sample (b) MLAC-525, (c) MLAC-625, (d) MLAC-725; energy-dispersive X-ray spectra of (e) carbonized DML sample (f) MLAC-525, (g) MLAC-625, (h) MLAC-725.

SEM images are frequently used to illustrate how raw materials are transformed into activated carbons by applying thermal or chemical treatments that modify the surface morphology of the raw materials.^27^ During the high-temperature treatment, activating agents like KOH can play a crucial role in corroding the carbon skeleton, which helps in the development of pores. Potassium vapours have the ability to penetrate carbon-based materials, resulting in swelling, disturbance of the carbon microstructure, and the creation of new pores.^28^ During the pyrolysis process, a significant quantity of small molecules like CO, CO_2_, C_*x*_H_*y*_, *etc.*, are liberated from inside the samples, which can also aid in the generation of pore structure. The SEM photographs in Fig. 2b–d demonstrate the three-dimensional pore architectures of the MLAC samples. Many efficient electrochemical sites are available when pores are present because they can buffer the electrolyte, helps in ion diffusion, and enable quick ion transport to the inner surface.^25^ As a result of its highly porous nature, MLAC is anticipated to provide superior charge accumulation when employed as electrodes for supercapacitors.

Morphological and physicochemical characteristics of activated carbon, such as area of surface, pore architectures, functional groups on the surface, and also the percentage of elemental compositions, are influenced by the pyrolysis temperature.^29^ Several possible pore structures were observed in the SEM image (Fig. 2a) of a carbonized sample. Additional pore creation and widening occurred at higher temperatures, resulting in an increase in surface area after pyrolysis with KOH. Prior to pyrolyzation, the carbonized sample exhibited a composition of 66.02% carbon and 33.98% oxygen. Subsequently, with an increase in the pyrolysis temperature from 525 °C to 725 °C, the percentage of carbon in the MLAC samples notably increased from 84.2 to 91.1, with a decline in the percentage of oxygen from 15.8 to 8.9, respectively. Hence, the pyrolysis temperature significantly impacted the oxygen–carbon (O/C) ratios of MLAC, as the ratio decreased from 0.19 to 0.10 with the increase in temperature. The upsurge in percentage carbon content in the X-ray energy dispersive spectroscopy report (Fig. 2e–h) of MLAC at higher temperatures was a reflection of the degree of carbonization, while the reduction in percentage oxygen content was probably a result of dehydration processes, the breakdown of oxygenated bonds, and the release of gaseous or low molecular weight by-products containing hydrogen and oxygen.^30^

**Fig. 3:**
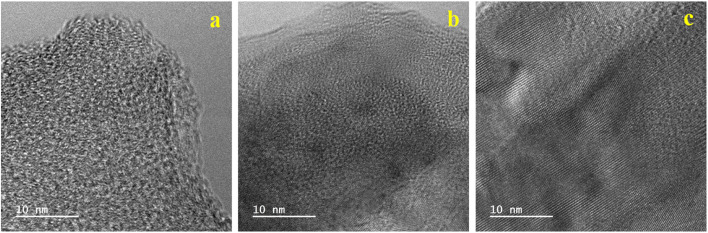
TEM images of (a) MLAC-525, (b) MLAC-625, (c) MLAC-725.

TEM analysis was also performed to obtain more topographical and morphological characteristics of the MLAC samples, and the images are illustrated in Fig. 3. The TEM images unambiguously reveal the coexistence of amorphous and graphitic features within the MLAC samples. Additionally, there is a discernible progression in the formation of ordered structures or graphitic layer-like arrangements from MLAC-525 to MLAC-725, suggesting a noticeable transition or evolution in the arrangement of the MLAC samples. This observation is not only visually evident but also finds support in the analyses conducted using both X-ray diffraction (XRD) and Raman spectroscopy. Hence, the correlation between TEM imagery and the results from XRD and Raman spectroscopy further strengthens our understanding of the structural changes occurring within the MLAC samples, consistently highlighting the evolving nature of the MLAC samples.

**Fig. 4:**
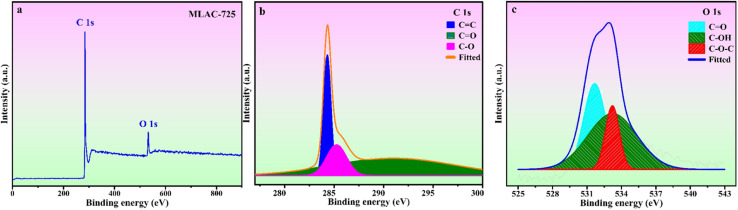
(a) XPS survey spectra & deconvoluted (b) C 1s (c) O 1s spectra of MLAC-725.

The surface chemical composition analysis of MLAC-725 was conducted through XPS measurements, as illustrated in Fig. 4. Fig. 4a, there are two noticeable peaks observed around ∼282.42 and ∼530.42 eV, which can be ascribed to carbon (C 1s, constituting 92.0% of the mass) and oxygen (O 1s, constituting 8.0% of the mass), respectively. The detailed C 1s spectra at higher resolution were analysed, resulting in three distinct peaks (Fig. 4b). These peaks can be attributed to sp^2^-bonded carbon (284.4 eV), C–O (286.4 eV), and C=O (288.8 eV). Furthermore, the high-resolution XPS analysis of O 1s spectra (Fig. 4c) revealed three peaks at 531.6, 532.6, and 533.6 eV, corresponding to C=O, C–OH, and O–C–O, respectively.^25^

**Fig. 5:**
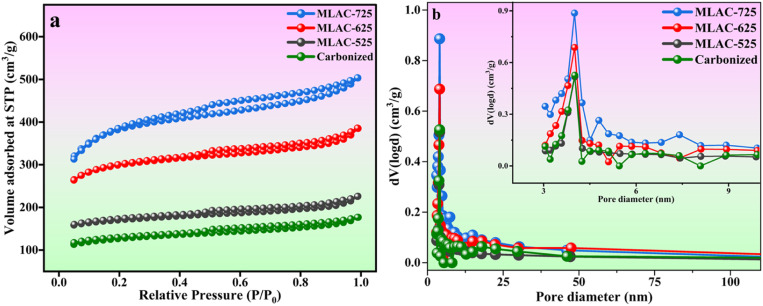
(a) N_2_ adsorption–desorption isotherms; (b) pore size distribution curves (inset: zoomed-in graph).

Along with its structural and morphological characteristics, the specific area of surface, total pore volume, and pore diameter of activated carbon are also important factors determining its adsorption capability for energy storage technologies.^31^Fig. 5 displays the N_2_ adsorption–desorption isotherms and pore size distribution for samples of MLAC-525, MLAC-625, and MLAC-725. According to Fig. 5a, all MLAC materials exhibit a classic type IV curve with a distinct H4 type hysteresis loop, indicating the presence of micropores and mesoporous, supported by the pore size distribution curves in Fig. 5b.^32^ Similar pore size distribution patterns were seen in all of the samples, and the peaks appeared nearly at 4 nm. According to their isotherms and pore size distributions, substantial surface areas of all MLAC are primarily due to the presence of a massive number of mesopores. After the carbonization of DML with FeCl_3_ at 400 °C, the formation of pore structures initiates, leading to an increase in surface area that eventually reaches 404.64 m^2^ g^−1^. Furthermore, the specific surface area (SSA) of synthesized MLAC samples rises from 534.74 to 1232.63 m^2^ g^−1^ (Table 1) with increasing experimental temperature from 525 °C to 725 °C. The carbon structure continues to hydrolysis at a higher rate as the temperature increases, leading to the development of additional micropores and the formation of mesopores, which result in an increased surface area.^33^ The increased SSA of activated carbon improves its capacity to hold effective charges.

**Table 1:** Textural parameters of MLAC

Sample	BET-SSA (m^2^ g^−1^)	Total pore volume^a^ (cm^3^ g^−1^)	Mean pore diameter (nm)
Carbonized sample	404.64	0.088	4.0133
MLAC-525	534.74	0.095	4.0102
MLAC-625	945.97	0.151	4.0055
MLAC-725	1232.63	0.199	4.0043

a Total pore volumes were determined at *P*/*P*_0_ = 0.99.

### 3.2. Electrochemical performance of electrodes

**Fig. 6:**
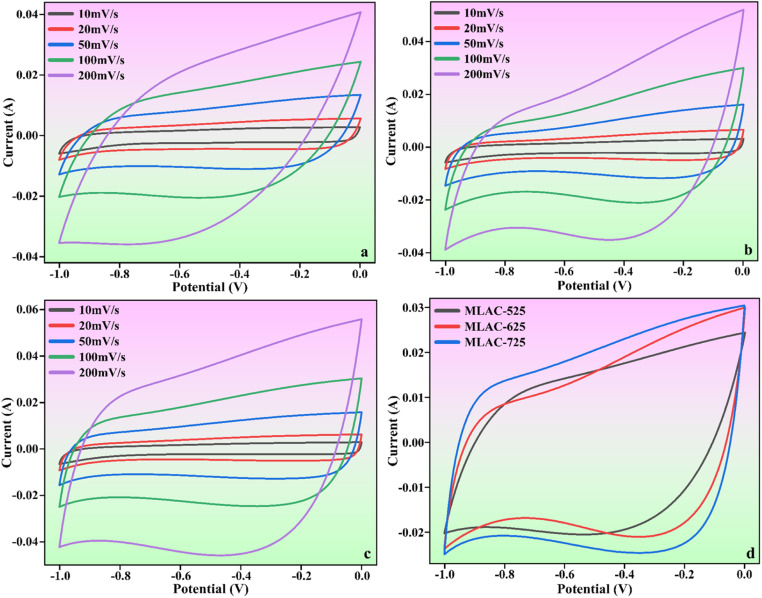
CV curves of (a) MLAC-525, (b) MLAC-625, (c) MLAC-725 (d) all the electrode materials at the scan rate of 100 mV s^−1^.

The findings and analyses from various structural and morphological studies indicated that MLAC-725, with its high BET surface area and highly ordered mesoporous structure, was the best-suited electrode material in all MLAC samples. To measure the electrochemical performances of MLAC-525, MLAC-625, and MLAC-725 were assessed as electrodes for supercapacitors in a two-electrode cell configuration using an aqueous 6 M KOH solution as an electrolyte. Fig. 6a–c shows the relevant electrochemical experiment findings of each MLAC electrode at different scan rates ranging from 10 mV s^−1^ to 200 mV s^−1^. Further, Fig. 6d compares the capacitive performances of all the samples at the same scan rate of 100 mV s^−1^ to identify the electrochemical benefits. Nearly rectangular voltammograms were obtained in CV studies, which reveals the typical characteristic of classic double-layer capacitive electrode behaviour even when operating at a high scan rate of 200 mV s^−1^.^34^ Compared to the MLAC-525 and MLAC-625 electrodes, the rectangular area of the MLAC-725 electrode on the CV curve was significantly greater, demonstrating the superior capacitive performance of the electrode. Additionally, the impacts of significant levels of developed graphitization, a well-established pore structure, and increased surface area in MLAC-725 materials result in improved capacitive characteristics.^35^

**Fig. 7:**
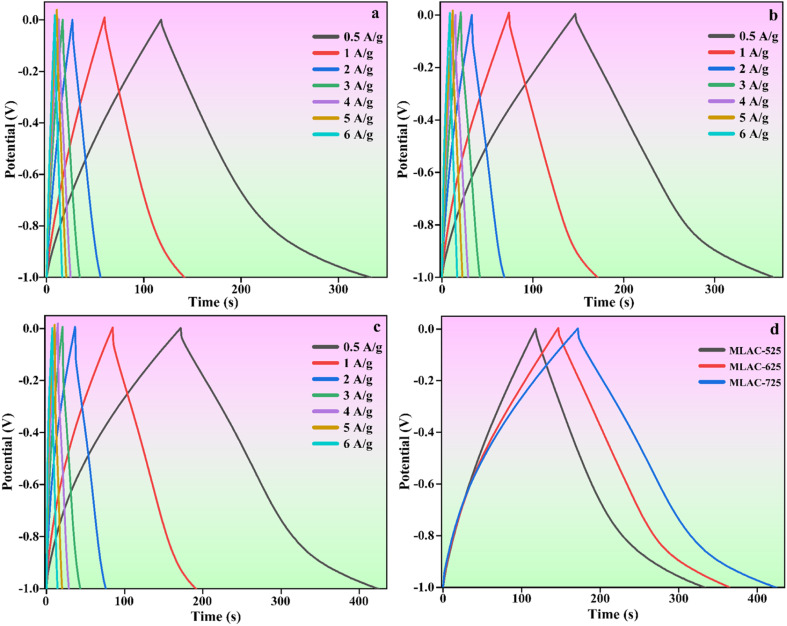
GCD curves of (a) MLAC-525, (b) MLAC-625, (c) MLAC-725 (d) all the electrode materials at the current density of 0.5 A g^−1^.

Further, galvanostatic charge–discharge (GCD) experiments were performed at different current densities ranging from 0.5 to 6 A g^−1^ to compute the specific capacitance of the MLAC materials. The typical findings are presented in Fig. 7. The fact that all of the charge and discharge curves have about symmetrical forms suggests that the material used for the electrode has remarkable electrochemical reversibility, and the relatively symmetric shapes of the galvanostatic charge–discharge (GCD) plots confirm their double-layer capacitor-like behaviour. At a current density of 0.5 A g^−1^, the calculated specific capacitance of MLAC-725 reached 521.65 F g^−1^, higher than the 444.90 F g^−1^ of MLAC-625 and the 440.82 F g^−1^ of MLAC-525. Because of the results of both physical and chemical activation, MLAC-725 has a significantly higher BET surface area, offering plenty of active sites for energy storage. Thus, it can be assumed that the amount of surface area significantly contributed to improving the material's capacitance by increasing the accumulation of ions at the junction of the electrode and the electrolyte.^36^ In a hierarchically porous graphitic texture, mesopores operate as easy paths for the ion to enable quick ion transmission, macropores act as ion-buffering reservoirs, and the graphitic structure offers a conductive system for swift charge movement. The combined presence of amorphous and graphitic carbon structures in MLAC-725 might have boosted wettability and increased electrical conductivity, which would be advantageous for capacitance enhancement.^25^Table 2 compares this work with a few reported results.

**Table 2:** Comparison of electrochemical performance of MLAC-725 with other reported materials

Carbon precursor	Electrolyte	Current density (A g^−1^)	Capacitance (F g^−1^)	Reference
*Mangifera indica* leaves	6 M KOH	0.5	521.65	This work
Human hair	6 M KOH	0.5	445.0	37
*Trichoderma* spent	6 M KOH	0.5	409.7	38
*Albizia* flowers	6 M KOH	0.5	406.0	39
*Auricularia*	6 M KOH	0.5	374.0	40
*Helianthus* pallet	6 M KOH	0.5	357.0	41
Oil-tea seed shell	6 M KOH	0.5	350.2	42

**Fig. 8:**
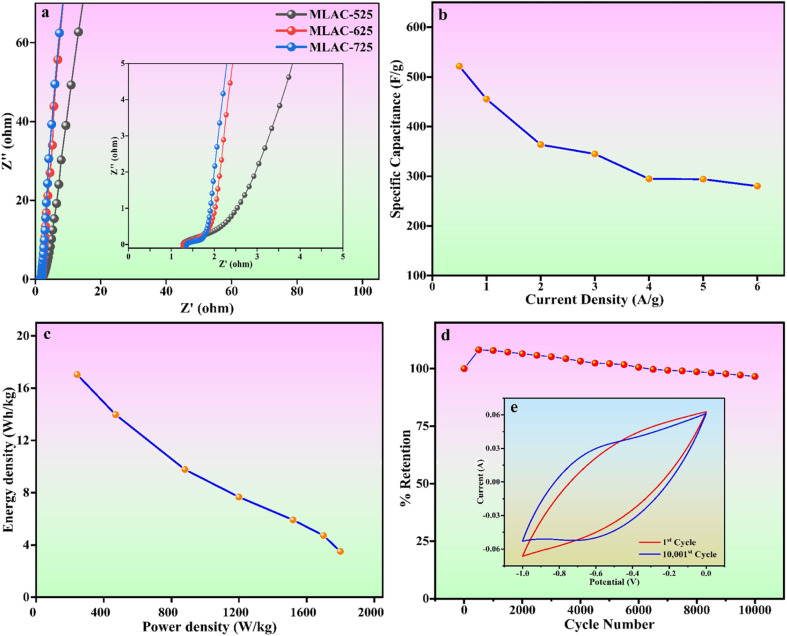
(a) Nyquist plots (inset: zoomed-in graph of the high-frequency region) (b) specific capacitance *versus* current density plot (c) Ragone plot (d) cyclic stability (e) 1^st^ and 10 001^st^ CV cycle.

Fig. 8a displays the Nyquist plot of the MLAC-525, MLAC-625, and MLAC-725 electrodes over the frequency range of 10 mHz to 100 kHz. In comparison, the MLAC-725 electrode has a very steep slope in the low-frequency zone and a comparatively small semi-circle radius in the high-frequency region. This indicates a faster ion diffusion, migration rate, and low charge transfer resistances suggesting the presence of a large number of micropores and a more organized microstructure in the MLAC-725.^38,43^ This could be the reason for the significant increase in the ion/charge conductivity inside the active carbon material to improve the rate of capacitive performance.^44^Fig. 8b displays a graph comparing specific capacitance to current density, while Fig. 8c illustrates Ragone plots. Both plots are based on the GCD results. The maximum energy density of MLAC-725 was determined to be 17.04 W h kg^−1^ at a power density of 242.50 W kg^−1^. Additionally, the cyclic stability of the fabricated electrodes was assessed, considering the importance of prolonged material stability in energy storage devices that undergo repeated cycles. The initial decrease in capacitance followed by an increase in subsequent cycles, observed in a cyclic stability study of a supercapacitor, is a common phenomenon. This behaviour can often be attributed to the activation and conditioning of the electrode materials during the initial cycles. In the early cycles, activation processes such as electrolyte penetration, ion migration, rearrangement of ions, surface restructuring or reorientation, and the formation of a stable interface between the electrode material and the electrolyte may take place, causing an initial decrease in capacitance. As the cycling continues, these processes stabilize, leading to an enhancement in the supercapacitor's performance and an eventual increase in capacitance. This phenomenon is part of the electrochemical processes in the initial stages of supercapacitor operation. The assembled capacitor exhibited exceptional long-term stability, retaining approximately 96.60% of its capacitance after 10 001 cycles at a scan rate of 500 mV s^−1^ in a 6 M KOH solution (Fig. 8d). The inset, *i.e.*, Fig. 8e presents a typical cyclic voltammogram for the 1^st^ and 10 001^st^ cycles, demonstrating consistent shapes with minimal deviation, indicative of outstanding capacitance characteristics. All of these superior electrochemical performances underscore the potential of the material as a supercapacitor electrode, derived from biomass waste.

## 4. Conclusions

In order to create a greener environment, it is suggested in this study that effective regulation may be used to develop efficient porous carbon compounds from waste biomass, which can then be used in energy storage devices like supercapacitors. The dead *Mangifera indica* leaves-derived activated carbons showed high specific surface areas, large pore volumes, suitable pore size distributions, and a lot of surface functionalities. The impacts of significant levels of developed graphitization, a well-established pore structure, and increased surface area in MLAC-725 materials result in improved capacitive characteristics. The combined presence of amorphous and graphitic carbon structures in MLAC-725 might have boosted wettability and increased electrical conductivity, which would be advantageous for capacitance enhancement. The highest electrochemical performances, *i.e.*, the maximum specific capacitance of 521.65 F g^−1^ at a current density of 0.5 A g^−1^, were attained when the specific surface area of the MLAC-725 was raised to 1232.63 m^2^ g^−1^. Significantly, it achieved an energy density of 17.04 W h kg^−1^ at a power density of 242.50 W kg^−1^. Furthermore, it demonstrated remarkable electrochemical cyclic stability by retaining 96.60% of its initial capacity even after undergoing 10 001 cycles at a scan rate of 500 mV s^−1^. The excellent electrochemical capabilities were obtained compared to other carbons reported (Table 2) to be made from biomass when these carbons were used as the material for the electrode in supercapacitors and other energy storage devices. As a result, this research effectively demonstrates the utilization of waste biomass resources to produce environmentally beneficial energy storage devices, highlighting a promising pathway towards a more sustainable and greener environment. Moreover, it presents substantial possibilities for the extensive adoption of advanced renewable energy solutions at a broader level.

## Author contributions

Mr Shreeganesh Subraya Hegde – conceived the original idea, conceptualization, experimental design, methodology, measurements, calculations, visualization, interpretation of results, validation, formal analysis, data curation, investigation and literature study, writing – original draft, review & editing, Dr B. Ramachandra Bhat – supervision, laboratory facility, review & editing.

## Conflicts of interest

The authors declare that they have no known competing financial interests or personal relationships that could have appeared to influence the work reported in this paper.

## Supplementary Material

RA-014-D3RA08910J-s001
