# Supplementary material for: Sustainable energy storage: Mangifera indica leaf waste-derived activated carbon for long-life, high-performance supercapacitors
Source: RSC Adv. 2024 Mar 7;14(12):8028–38. doi: 10.1039/d3ra08910j (PMC10918766; doi:10.1039/d3ra08910j)
Supplement: RA-014-D3RA08910J-s001 [file RA-014-D3RA08910J-s001.pdf]

Electronic Supplementary Material (ESI) for RSC Advances.
This journal is © The Royal Society of Chemistry 2024

Sustainable Energy Storage: Mangifera Indica Leaf Waste-Derived Activated Carbon for Long-Life, High-Performance Supercapacitors

Shreeganesh Subraya Hegde*, Badekai Ramachandra Bhat*

Catalysis and Materials Chemistry Laboratory, Department of Chemistry, National Institute of Technology Karnataka, Surathkal, Mangalore, 575025, Karnataka, India

* Corresponding : hegdeshreeganesh@gmail.com (ORCID(S): 0000-0003-2678-7481)

ram@nitk.edu.in (ORCID(S): 0000-0002-8169-1055)

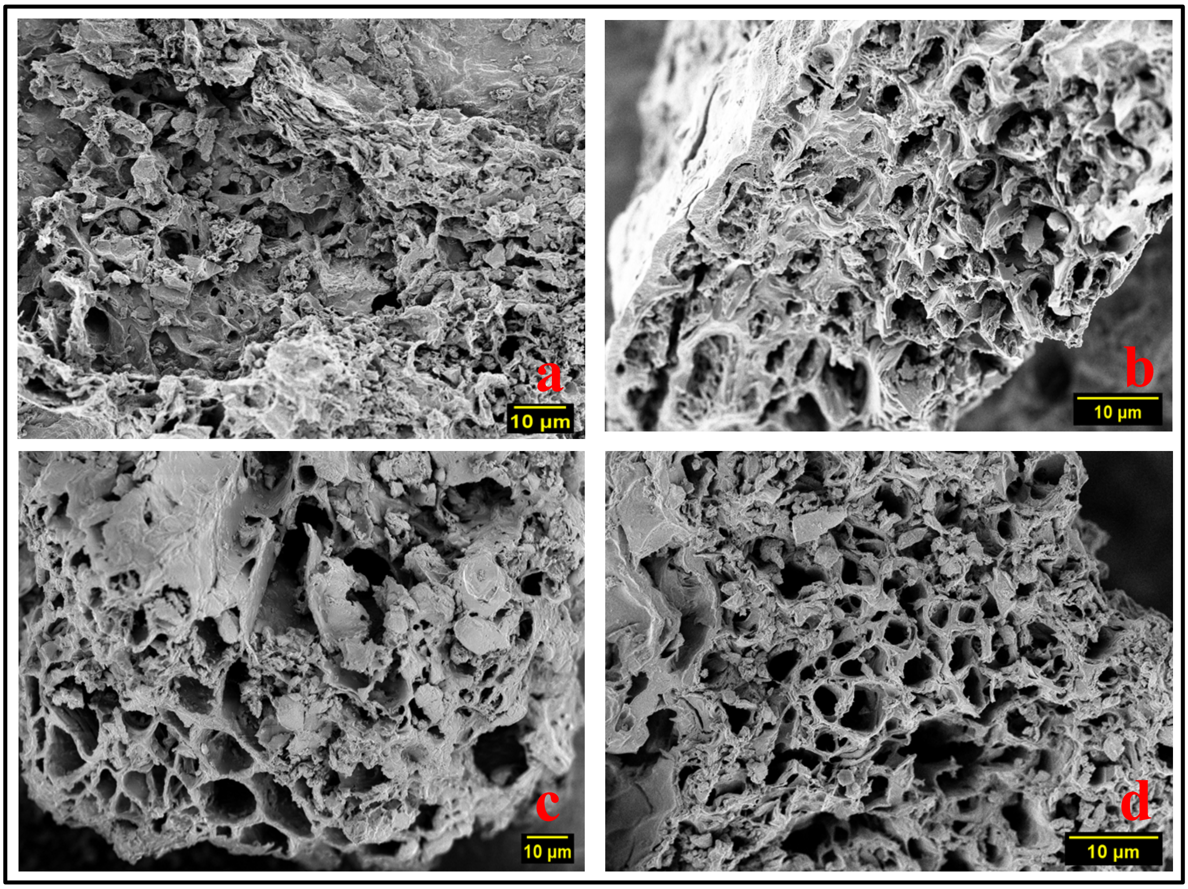

Figure 1. FESEM images of (a)Carbonized DML sample (b) MLAC-525, (c) MLAC-625, (d) MLAC-725 at low power/voltage.
